# Developing a hetero-intelligence methodological framework for sustainable policy-making based on the assessment of large language models

**DOI:** 10.1016/j.mex.2024.102707

**Published:** 2024-04-14

**Authors:** Eva M. Buitrago-Esquinas, Miguel Puig-Cabrera, José António C. Santos, Margarida Custódio-Santos, Rocío Yñiguez-Ovando

**Affiliations:** a Faculty of Economics and Business Sciences, Universidad de Sevilla, Spain; b Research Centre for Tourism, Sustainability and Well-being (CinTurs), Universidade do Algarve, Faro, Portugal

**Keywords:** Sustainable planning and policy, Hetero-intelligent performance testing, Conversational generative AI, Human intelligence, Large language models, ChatGPT, hetero-intelligence methodological framework for sustainable policy-making

## Abstract

This work delves into the increasing relevance of Large Language Models (LLMs) in the realm of sustainable policy-making, proposing an innovative hetero-intelligence framework that blends human and artificial intelligence (AI) for tackling modern sustainability challenges. The research methodology includes a hetero-intelligence performance test, which juxtaposes human intelligence with AI in the formulation and implementation of sustainable policies. After testing this hetero-intelligence methodology, seven steps are rigorously described so that it can be replicated in any sustainability planning related context. The results underscore the capabilities and limitations of LLMs, underscoring the critical role of human intelligence in enhancing the efficacy of hetero-intelligence systems. This work fulfils the need of a rigorous methodological framework based on empirical steps that can provide unbiased outcomes to be integrated into sustainable planning and decision-making processes.

• Assesses LLMs’ limitations and capabilities regarding sustainable planning issues
• A replicable methodology is proposed based on the combination of both human and artificial intelligence
• It proposes and systematises the integration of a hetero-intelligent approach into the formulation of sustainability policies to be more efficient and effective

## Graphical abstract

**Figure:**
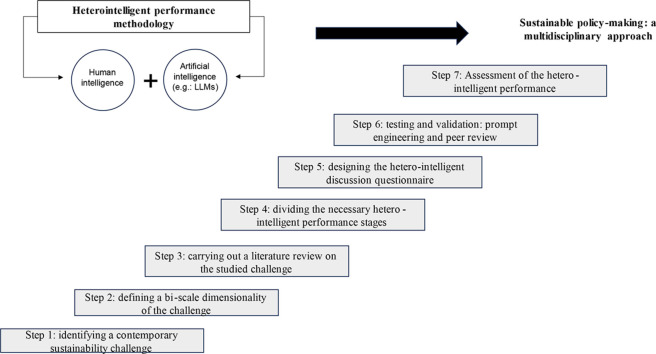

Specifications table

**Table:** 

Subject area:	Earth and Planetary Sciences
More specific subject area:	Sustainable Planning
Name of your method:	hetero-intelligence methodological framework for sustainable policy-making
Name and reference of original method:	None
Resource availability	Large Language Models, such as ChatGPT or similar. For example: chat.openai.com

## Method details

A consensus is emerging on the significant impact of large language models (LLMs) in reshaping societal norms, individual interactions, and the competencies required for future professional success [1]. LLMs, defined as 'AI tools utilizing extensive recurrent neural networks, are trained on substantial data pools to produce text resembling human output' ([2], p. 1549). These models adapt and improve through their use, leading to novel human-AI collaboration scenarios, which we refer to as hetero-intelligence systems. This concept integrates hybrid intelligence—'systems that synergize human and AI strengths to outperform their individual capabilities and learn reciprocally' ([3], p. 3)—with Breakspear's intelligence framework, which emphasizes predictive and analytical abilities in problem resolution [4]. Our proposed hetero-intelligence model merges human and AI intellects to proactively address societal challenges with deep understanding and foresight, advocating for human oversight in these integrations for optimal outcomes.

LLMs are gaining traction across various sectors, illustrated by their applications in medical diagnostics [5] and academic writing [6]. Research on LLMs in the realm of sustainability and policy design is in its infancy, often limited to theoretical or subjective assessments. Present studies mainly explore potential LLM applications in addressing sustainable development issues [[1], [7], [8], [9]], but the scope of these explorations is frequently narrow. Scholars concur on the necessity for comprehensive research into the impact of LLMs on sustainable progress and strategic planning [[10], [11], [12]]. However, there's a noticeable dearth of empirical research into LLMs' role in sustainable policy formulation [8,9], a gap we aim to address.

The main goal of this work is to introduce a tested innovative hetero-intelligence methodological framework that merges human intelligence with AI for specialized analysis and decision-making in sustainability issues.

The integration of AI into the tourism sector constitutes a significant stride towards using innovative methods to make the most of technology's ability to contribute directly to policy-making processes. This research's pioneering methodology concentrated on contrasting artificial and human intelligences to guide conversational flow and assess how a hetero-intelligence approach can be integrated into sustainable planning based on the assessment of LLMS.

**Fig. 1:**
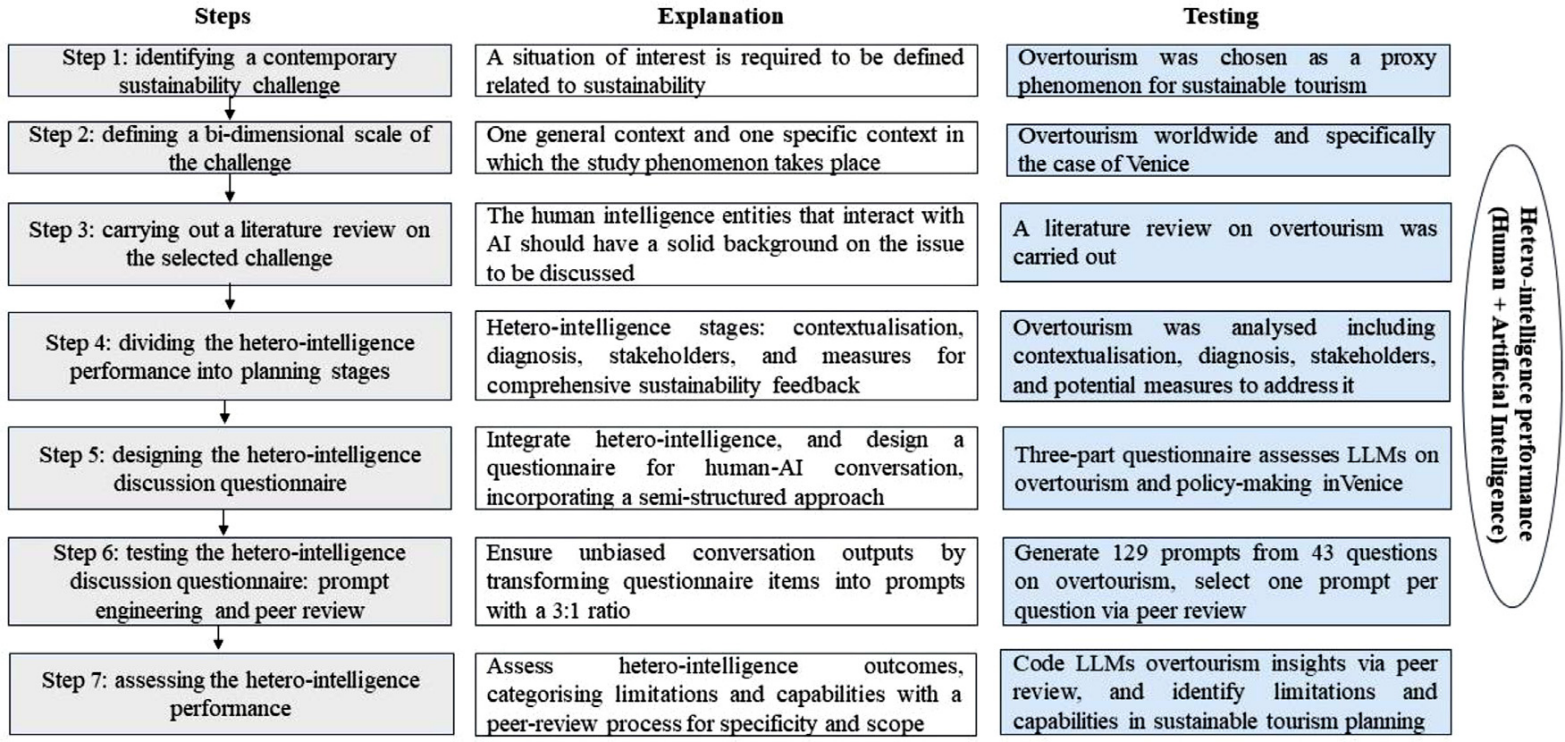
Methodological guidelines to apply a hetero-intelligent methodology for policy-making regarding a sustainable challenge. ***Source:*** Buitrago-Esquinas et al. (related published article under review).

The proposed hetero-intelligence method of sustainability planning and policymaking was built upon seven steps that considered other previously consolidated methodologies. These steps were rigorously followed as presented in Fig. 1:

- *Step one: Identifying a contemporary sustainability challenge.* This step involves delineating a scenario that encapsulates a significant sustainability concern, which may encompass a specific problem, prevailing issue, or notable event, as highlighted by Crabolu et al. [13]. Such challenges should be recognized and addressed by decision-makers in specific contexts. In this case, the phenomenon of overtourism was identified as a representative challenge for testing our methodology, given its prominence as a critical and pressing concern in contemporary sustainable tourism management.
- *Step two: Defining a bi-dimensional scale of the challenge*. A critical challenge in utilizing AI-based tools for policymaking in sustainability issues lies in their ability to interpret and respond to diverse contexts [[1], [7], [14]]. To effectively evaluate the applicability of these AI tools, it's essential to analyze sustainability challenges using a bi-dimensional framework, encompassing both broad and specific scenarios. The present research chose overtourism on a global scale and a well-known case study of this phenomenon, namely, Venice [[15], [16]]. Thus, the capabilities and limitations of AI and human intelligence interactions are contrasted while focusing on a specific sustainable tourism challenge for a general and a specific context.
- *Step three: Carrying out a literature review on the selected challenge*. For the hetero-intelligence performance test to be effective, the human intelligence interacting with the AI must possess a thorough understanding of the subject matter. This foundational knowledge is crucial for ensuring meaningful and productive collaboration between human and artificial intelligence, leading to successful outcomes in the analysis and resolution of complex issues. In this case, the most updated research was collected to compose a proper state-of-the-art summary of overtourism in general and in the chosen case study (e.g. Venice). Fifty relevant papers were chosen to complement previously acquired knowledge about overtourism.
- *Step four: Dividing the hetero-intelligence performance into planning stages*. This phase entails outlining the various stages where hetero-intelligence performance will be integrated into sustainable planning. The initial stage focuses on contextualizing the issue at hand, understanding its intrinsic factors and structure. Another critical stage involves engaging stakeholders, identifying the diverse actors involved, and understanding their unique interests and roles in the sustainable challenge. Additionally, a crucial stage is evaluating the effectiveness of LLMs in formulating strategies and decision-making processes. This includes brainstorming tailored measures to address sustainability challenges and adapting these solutions to the specific contexts under study. Adopting a 360° approach, considering all these stages, ensures a more comprehensive evaluation and feedback throughout the methodology, as demonstrated in the testing of the current proposed method.
- *Step five: Designing the hetero-intelligence discussion questionnaire.* This phase entails creating a questionnaire modeled on a semi-structured interview approach [17]. It is essential to incorporate open-ended questions, as this format compels the AI to generate more comprehensive responses. The development process encompasses initial pilot testing, validation of the content, and methodological triangulation [18]. The questions in the questionnaire should align with the bi-dimensional scale applied and encompass all aspects of the focus area. Furthermore, the questionnaire's design should be informed both by an extensive review of relevant literature and the nuanced insights of the human experts involved. For instance, during the testing of the methodology, the questionnaire was divided into three sections with a total of 43 open-ended questions. The first section was a self-diagnosis in which the selected LLM was asked about its capabilities and limitations within sustainable tourism planning in general terms. The second section focused on aspects related to diagnosis of overtourism. The last section was related to the formulation of policies to manage overtourism in a destination.
- *Step six: Testing the hetero-intelligence discussion questionnaire: prompt engineering and peer review*. Prompt design can condition the output obtained from human intelligence's conversation with an LLM. For this reason, each item of the current questionnaire should be transformed into a prompt to be tested by the chosen LLM. A 3:1 ratio is applied, thereby comparing two alternative questions in addition to the original item. In the present case, 129 prompts were made out of the 43 original questions. The main goal of this step is to choose 1 prompt out of 3 for each question and discard the alternative versions of that question. This prompt engineering process [19] had been previously validated by a peer review process conducted by the researchers involved in the current research, which consisted of each reviewer having the same output in parallel with an LLM to select 1 out of 3 prompts per question. Thus, each output coming from the 129 prompts is compared by the researchers. Finally, the results were discussed by the authors so that the 43 final prompts were chosen as those producing most complete output from the selected LLM. The sixth step ends with the finalised version of the questionnaire that will guide the conversation between the human intelligence and AI within the sustainability challenge under study.
- *Step seven: Assessing the hetero-intelligence performance*. In the final stage, the focus shifts to analyzing the results obtained from the hetero-intelligence dialogue, specifically evaluating the strengths and weaknesses manifested during the interaction. This evaluation hinges on the nature and extent of the queries posed. To achieve this, a subsequent round of peer reviews is conducted, aiming to categorize the responses from each query in terms of their broad or specific efficacies and shortcomings. Employing such a methodology to delineate the functional boundaries of LLMs [1] is essential. It enables the fusion of theoretical understanding and practical insights from human experts with the computational output of AI, thus facilitating a nuanced understanding of the subject matter. In the current case, overtourism phenomenon was discussed with a ChatGPT-4 version developed by OpenAI because of this tool's similarities with other LLMs.

After testing the proposed methodology, the results reveal that a hetero-intelligence approach can be useful within sustainable planning once the capabilities and limitations of both human intelligence and AI are properly identified. LLMs, as an AI application, can become a powerful tool for diagnosing sustainable issues and proposing measures to address sustainability-related challenges, such as overtourism. Also, this work fulfils the need of a rigorous methodological framework based on empirical steps that can provide unbiased outcomes to be integrated into sustainable planning processes.

Although the hetero-intelligence framework was tested with ChatGPT-4, it is adaptable to other existing LLM-based solutions (e.g.: Bard), as they have a similar architecture. Consequently, while the proposed framework aims to facilitate unbiased outcomes through the synergistic partnership of AI and human intelligence, evaluation and customization in the application of the framework to different LLMs should be considered to mitigate inherent biases and tailor the approach to the specific characteristics and capabilities of each model.

This methodology is designed to integrate human intelligence as a means to be combined with AI and assure a more effective policymaking process guaranteeing unbiased. One of the main contributions of the human intelligence is based on the deep understanding about the issue under study, including also ethical, cultural and intuitive expertise. Therefore, while the integration of human and AI intelligence seeks to enhance objectivity and decision-making, guaranteeing absolute unbiased outcomes remains a complex challenge. Therefore, the methodology emphasizes the critical role of human intelligence in identifying, evaluating, and mitigating potential biases inherent in AI-generated solutions for more balanced and informed policy outcomes.

In conclusion, the proposed hetero-intelligence methodology holds significant potential for addressing a wide array of sustainability challenges across both public and private sectors. Its applicability ranges from urban planning and environmental conservation to corporate sustainability and social responsibility initiatives. The methodology has a flexible design that makes possible to be tailored to the specific needs and contexts of different sectors, thereby, becoming a versatile tool for integrating AI into sustainable decision-making processes.

## Ethics statements

None.

## CRediT authorship contribution statement

**Eva M. Buitrago-Esquinas:** Conceptualization, Formal analysis, Methodology, Validation, Writing – original draft. **Miguel Puig-Cabrera:** Conceptualization, Formal analysis, Methodology, Validation, Writing – original draft. **José António C. Santos:** Conceptualization, Formal analysis, Methodology, Validation, Writing – original draft. **Margarida Custódio-Santos:** Conceptualization, Formal analysis, Methodology, Validation, Writing – original draft. **Rocío Yñiguez-Ovando:** Conceptualization, Formal analysis, Methodology, Validation, Writing – original draft.

## Declaration of competing interest

The authors declare that they have no known competing financial interests or personal relationships that could have appeared to influence the work reported in this paper.

## Data Availability

Data will be made available on request.
